# 5-HT1A Receptors Alter Temporal Responses to Broadband Vocalizations in the Mouse Inferior Colliculus Through Response Suppression

**DOI:** 10.3389/fncir.2021.718348

**Published:** 2021-08-27

**Authors:** Arianna Gentile Polese, Sunny Nigam, Laura M. Hurley

**Affiliations:** ^1^Department of Cell and Developmental Biology, University of Colorado Anschutz Medical Campus, Aurora, CO, United States; ^2^Department of Biology, Program in Neuroscience, Indiana University Bloomington, Bloomington, IN, United States; ^3^Department of Neurobiology and Anatomy, McGovern Medical School, The University of Texas Health Science Center at Houston, Houston, TX, United States; ^4^Department of Physics, Indiana University Bloomington, Bloomington, IN, United States

**Keywords:** serotonin receptor, 5-HT1A, inferior colliculus, auditory, vocalization

## Abstract

Neuromodulatory systems may provide information on social context to auditory brain regions, but relatively few studies have assessed the effects of neuromodulation on auditory responses to acoustic social signals. To address this issue, we measured the influence of the serotonergic system on the responses of neurons in a mouse auditory midbrain nucleus, the inferior colliculus (IC), to vocal signals. Broadband vocalizations (BBVs) are human-audible signals produced by mice in distress as well as by female mice in opposite-sex interactions. The production of BBVs is context-dependent in that they are produced both at early stages of interactions as females physically reject males and at later stages as males mount females. Serotonin in the IC of males corresponds to these events, and is elevated more in males that experience less female rejection. We measured the responses of single IC neurons to five recorded examples of BBVs in anesthetized mice. We then locally activated the 5-HT1A receptor through iontophoretic application of 8-OH-DPAT. IC neurons showed little selectivity for different BBVs, but spike trains were characterized by local regions of high spike probability, which we called “response features.” Response features varied across neurons and also across calls for individual neurons, ranging from 1 to 7 response features for responses of single neurons to single calls. 8-OH-DPAT suppressed spikes and also reduced the numbers of response features. The weakest response features were the most likely to disappear, suggestive of an “iceberg”-like effect in which activation of the 5-HT1A receptor suppressed weakly suprathreshold response features below the spiking threshold. Because serotonin in the IC is more likely to be elevated for mounting-associated BBVs than for rejection-associated BBVs, these effects of the 5-HT1A receptor could contribute to the differential auditory processing of BBVs in different behavioral subcontexts.

## Introduction

Neuromodulatory neurons that arise outside of the auditory system and synthesize monoamines such as catecholamines and serotonin (Klepper and Herbert, 1991; Forlano et al., 2015; Nevue et al., 2016; Barr and Woolley, 2018; Ghahramani et al., 2018; Schofield and Hurley, 2018; Hurley, 2019; Petersen et al., 2020) can reconfigure auditory circuitry to modify the magnitude and timing of responses to acoustic stimuli (Hurley et al., 2004; Gittelman et al., 2013; Jacob and Nienborg, 2018; Hoyt et al., 2019; Sizemore et al., 2020). However, with some exceptions (e.g., Hurley and Pollak, 2005; Ikeda et al., 2015; Lee et al., 2018), studies of neuromodulatory effects on auditory responses have not examined how neuromodulators affect responses to the types of natural vocal signals that occur in conjunction with neuromodulatory release.

To explore this issue, we assessed the effects of manipulating the serotonergic system on the responses of mouse midbrain auditory neurons in the inferior colliculus (IC) to a type of vocalization with an established relationship to social behavior, and also to serotonin release. The vocalization type is an audible call made by mice in several different contexts. Most commonly known as “squeaks,” they have also been called low-frequency harmonic calls or broadband vocalizations (BBVs); the latter designations refer to the presence of prominent low-frequency harmonics that extend into the ultrasonic range (Grimsley et al., 2013; Lupanova and Egorova, 2015; Finton et al., 2017). During opposite-sex interactions, BBVs are predominantly produced by females (Wang et al., 2008a). Even within the opposite-sex context, females produce BBVs in different sub-contexts. During the initial investigative stages of opposite-sex interaction, females produce BBVs that correspond in number and time with kicks or lunges at males (Sugimoto et al., 2011; Finton et al., 2017). BBVs produced in this stage correspond to a smaller number of ultrasonic vocalizations (USVs) produced by males, and predict less male mounting of females in later stages of the interaction. When mounting does occur, however, BBVs are often produced as females are being mounted, and these BBVs may overlap in time with USVs (White et al., 1998; Finton et al., 2017). Males may respond to BBVs with relative attraction (Grimsley et al., 2013), or as if they constitute a signal of rejection (Hood et al., 2020), depending on the behavioral context.

Broadband vocalizations also correlate with activation of the serotonergic system within the IC, which is a hub for ascending and descending auditory pathways (Schofield and Cant, 1999; Coomes and Schofield, 2004; Loftus et al., 2010; Bartlett, 2013; Lesicko et al., 2016; Patel et al., 2017). In the IC of males interacting with females, serotonin measured voltametrically through carbon fiber electrodes is inversely correlated with the number of BBVs produced by their female social partners (Keesom and Hurley, 2016). Serotonin in the IC of males is thus higher when they experience a lack of rejection from females. The sources of serotonin to the IC are two distinct subpopulations of neurons within the dorsal raphe nucleus (DRN; Petersen et al., 2020). These subpopulations are involved in social aggression (Niederkofler et al., 2016), and show distinct responses to social context (Petersen et al., 2020). All of these findings suggest that BBVs are heard by males in different contexts, and that some of these contexts correspond to heightened serotonergic modulation. However, there is no understanding of how IC neurons respond to BBVs, or how serotonin influences the responses of IC neurons to BBVs.

Further complicating the situation is the fact that there are multiple types of serotonin receptor within the IC. Simply increasing serotonin in the IC typically causes a range of effects on the responses of IC neurons that vary across neurons, types of sound, and over time (Hurley and Pollak, 1999, 2005; Bohorquez and Hurley, 2009). In the current study, we therefore took the approach of looking at the effect of one type of receptor, the 5-HT1A receptor. This receptor type is commonly expressed across brain regions, and is prominently expressed in the IC (Thompson et al., 1994; Peruzzi and Dut, 2004; Smith et al., 2014). Many IC neurons respond to activation of the 5-HT1A receptor with suppression of sound-evoked responses (Hurley, 2006, 2007; Castellan Baldan Ramsey et al., 2010). With these points in mind, we took an experimental approach allowing the local pharmacological manipulation of 5-HT1A receptors in the IC of intact mice through iontophoresis of a 5-HT1A agonist and antagonist. This was accomplished in anesthetized mice, allowing for the stable recording of single neurons during iontophoresis. Anesthetized mice also show low endogenous levels of serotonin release (Hall et al., 2010). We found that BBVs evoke responses that are not selective at the level of individual IC neurons, but which show distinct patterns of spikes over time that vary across call types and neuron identities. Activation of 5-HT1A receptors suppresses responses to BBVs, and results in the loss of some response features but not others, altering BBV encoding.

## Materials and Methods

### Animals and Surgical Procedures

All procedures followed the NIH guidelines for the proper care and use of laboratory animals, and were approved by the Bloomington Institutional Animal Care and Use Committee. 77 single units were recorded from 15 adult male CBA/J mice (The Jackson Laboratory, Bar Harbor, ME, United States). Mice were anesthetized via brief exposure to isoflurane fumes, immediately followed by intraperitoneal injection of a drug mixture consisting of 120 mg/kg ketamine and 5 mg/kg xylazine. Ophthalmic ointment was applied to prevent the eyes from drying, and a depilatory cream was used to remove the hair on top of the head. The skin on the head was incised along the midline and reflected to each side, and adherent tissue was cleared from the surface of the skull. After steadying the head with a bite bar and ear bars, a hole was drilled in the skull over each IC. The dura was incised and cleared with a tungsten probe, and each hole was filled with silicon gel. A layer of glass beads was applied to the skull anterior to lambda with cyanoacrylate glue. The mouse was transferred to a custom stereotaxic device in a sound-attenuated chamber, and a post was affixed to the skull anterior to the drilled holes using dental cement. Body temperature was maintained between 36 and 37°C with a temperature regulation system (FHC, Bowdoinham, ME, United States). Throughout the experiment, supplemental doses of either the presurgical anesthetic mixture or ketamine alone were used to maintain the level of anesthesia.

### Electrodes and Recording Procedures

Responses of single cells were recorded extracellularly using high-resistance glass micropipettes (A-M Systems, Carlsborg, WA, United States), connected to a Dagan 2400 amplifier (Minneapolis, MN, United States) by a silver-silver chloride wire. Iontophoresis of drugs was done via a three-barreled pipette attached to the single-barreled recording pipette (single electrode blanks: 6010, three-barreled blanks: 6090; A-M Systems, Carlsborg, WA, United States). After three-barreled pipettes were pulled (A-M Systems; Stoelting 51210; Wood Dale, IL, United States), the tip was broken back to a diameter of 10–15 μm. The single-barrel recording pipettes were attached to the three-barrel pipette so that the recording pipette tip protruded 10–20 μm from the tip of the three-barrel pipette. Recording electrodes had a resistance of 8–20 MΩ under recording conditions. A dissecting microscope was used to visually position the assembled combination electrodes above the IC. The electrode was then lowered using a piezoelectric microdrive (Burleigh/EXFO Inchworm, Mississauga, ON, Canada) in increments of 1 μm. Search stimuli were tones corresponding to the approximate tonotopic location of the electrode (frequencies increase with increasing recording depth), and narrowband noise (NBN) with a bandwidth of 10 kHz, and a center frequency corresponding to the approximate tonotopic location. Electrodes were lowered until well-isolated action potentials (signal:noise ratio >10) responding to one of the search stimuli could be observed. Most neurons showed low levels of spontaneous activity, as previously reported for recording single neurons through glass micropipettes (Hurley and Pollak, 1999, 2005). Data collection for all stimuli began 10 ms prior to stimulus presentation. The responses of neurons to experimental stimuli were recorded before, during, and when possible after the iontophoresis of drugs or vehicle. Following data collection on a single neuron, current was ejected from the electrode to “kill” it; this was done to eliminate the possibility of recording from the same cell twice. After recording from a cell, the electrode was lowered until the next responsive cell was encountered. This was repeated until the electrode reached a depth at which cells did not respond to our vocal stimuli; at this point the electrode was raised out of the IC and repositioned to record from a new site. Spikes were passed through a spike signal enhancer (FHC, Bowdoinham, ME, United States) before being digitized through a data acquisition processor board (Microstar DAP5216A/626; Bellevue, WA, United States). Data was collected and stored for later analysis by the software package Batlab (Dr. Donald Gans, Kent State University).

Recordings were concentrated in the caudal and medial 2/3 of the IC based on landmarks including lambda and blood vessels (Hage and Ehret, 2003; Franklin and Paxinos, 2008). Electrode penetrations were centered approximately 1 mm caudal and medial to lambda and ranged up to 0.5 mm from this central point, targeting the central subdivision of the IC. Most neurons showed well-defined tuning curves. Additionally, most neurons were part of a clear tonotopic progression, with characteristic frequencies (CFs) extending from as low as 3 kHz at more dorsal electrode locations to as high as 55 kHz at more ventral locations. The location of the recording sites was confirmed in eight cases by iontophoretic deposition of neurobiotin (1% in 1M NaCl; Vector Laboratories, Burlingame, CA, United States) during the recording session using a dedicated single-barreled electrode, followed by intracardiac perfusion at the end of the experiment. After brain tissue was extracted, fixed in 4% formaldehyde and sectioned at 50 μm, sections of IC were incubated in fluorescein streptavidin (Vector Laboratories, Burlingame, CA, United States) and visualized under a fluorescence microscope (Nikon Ni-E, Nikon Instruments) at 515 nm. Neurobiotin-labeled cell bodies were observed in the central subdivision of the IC in all cases. However, since experiments consisted of multiple electrode penetrations, we cannot exclude the possibility that some neurons were located in the external nucleus as well.

### Drugs and Iontophoresis

Two drugs targeting the 5-HT1A receptor were used in this study: the agonist (±)-8-hydroxy-2-dipropylaminotetralin hydrobromide (8-OH-DPAT), and (3*R*)-3-(Dicyclobutylamino)-8-fluoro-3,4-dihydro-2*H*-1-benzopyran -5-carboxamide hydrochloride (NAD-299). All drugs were obtained from Tocris Bioscience (Ellisville, MO, United States). Drugs were dissolved in 10 mM NaCl at pH 4.5. Drugs were retained in two of the three iontophoresis barrels with a current between −10 and −25 nA, and ejected using a range of currents up to +75 nA. The third barrel was filled with 1 M NaCl and balanced the currents ejected through the drug barrels. The three barrels were connected to iontophoresis pump modules [Dagan ION-100 or Medical Systems NeuroPhore (Harvard Apparatus, Holliston, MA, United States)] via silver-silver chloride wires. Control solutions consisted of the vehicle of 1 M NaCl at pH 4.5, or of 1 M NaCl with 10 mM NaBr. The same sets of stimuli were presented during the pre-drug period, during drug treatment beginning 3–5 min after the onset of drug iontophoresis, and, when neural recordings could be held for long enough, 5–10 min after drug application ceased and retention current was re-applied. 5-HT1A receptors were iontophoretically manipulated for 59 neurons. In some cases multiple drugs were iontophoresed alone and/or in combination. In total, NAD-299 was applied to 40 neurons (alone: *n* = 35; +8-OH-DPAT: *n* = 25), and 8-OH-DPAT was applied to 46 neurons (alone: *n* = 46; +NAD-299: *n* = 25). For experiments assessing the selectivity of 8-OH-DPAT by comparing it to the effects of NAD-299 in the same neurons, we used the acoustic stimuli generating the most robust responses. For the 34 neurons in the specific experiments on pharmacological selectivity (*n* = 20 for DPAT versus NAD and 14 for vehicle), responses to Call 5 (see below) were used for 16 neurons, responses to other calls for two neurons, and responses to tones at best frequency for 16 neurons.

### Auditory Stimuli

Tone stimuli were generated by BATLAB software and were routed through a PA5 attenuator and FT-6 antialias filter (TDT, Alachua, FL, United States). Stimuli were played through a Vifa speaker, with an operational range from 1 to 120 kHz (Avisoft Bioacoustics, Glienicke/Nordbahn, Germany). Frequency tuning was measured by presenting tones of 20 ms in duration with rise and fall times of 0.5 ms across the range of frequencies that single cells were responsive to, from 10 dB below threshold to 30–50 dB above threshold at the CF. Depending on the bandwidth of the cell, the frequency intervals of the tones presented varied from 1 to 5 kHz. For neurons that did not respond to tones, narrowband noise (NBN) was used to estimate the CF, as the center frequency of a NBN with a 10 kHz bandwidth that evoked the most robust neural response.

**FIGURE 1:**
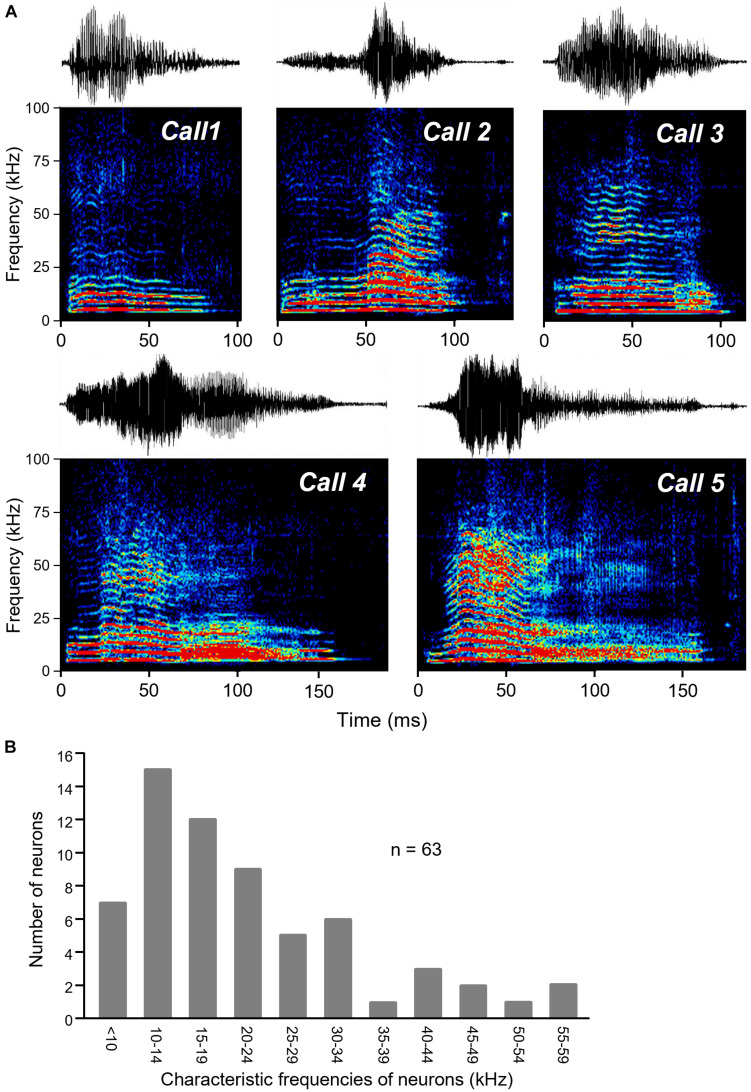
BBVs played to mice. **(A)** Oscillograms (top part of each panel) and spectrograms (lower part of each panel) of the five recorded BBVs used as stimuli for IC neurons. Calls varied in amplitude envelope as well as in duration, spectrotemporal structure, and whether deterministic chaos was present (in the latter portions of calls 3, 4, and 5). Colors represent relative intensity of specific call components. **(B)** Histogram of the characteristic frequencies (CF) of neurons responding to BBVs, which also responded to tones for estimation of CF.

Five different audible broadband vocalizations (BBVs, also “squeaks”) were used as stimuli. The BBVs were recorded from females during opposite-sex encounters with 16- bit resolution using a condenser microphone (CM16/CMPA; Avisoft Bioacoustics, Berlin, Germany; 200 kHz maximum range) and sound card (250 kHz sample rate, UltraSoundGate 116 Hb, Avisoft Bioacoustics). Calls were resampled using a custom Matlab script to match the output of the Microstar board. BBVs were chosen to represent a range of structural features characteristic of these call types (Figure 1; Lupanova and Egorova, 2015; Finton et al., 2017). BBVs varied in duration ranged in duration from 104.6 ms (Call 1) to 187.9 ms (Call 5). Calls also varied in their amplitude envelopes, spectrotemporal structure, and the presence and duration of deterministic chaos (DC: structured noise due to non-linear vocal fold vibration) versus harmonic structure. For example, Calls 3, 4, and 5 each contained a period of DC in the terminal portion of the call, with these segments being longer for Calls 4 and 5. Although BBVs have not been categorized in the same way that mouse USVs have been, variation in total duration and the relative duration of DC segments correspond to multiple behaviorally relevant characteristics (Finton et al., 2017). DC varies consistently and significantly across individual females, and females produce calls with a higher percent duration of DC when they are in estrus relative to diestrus. Relatively longer BBVs and BBVs with a higher relative DC duration are also more likely to occur when females are being mounted relative to when females are showing rejection of males. Measurements of responses to calls were made at 10–30 dB above the threshold for the lowest intensity evoking a response from any call.

### Data Analysis

Spike trains were recorded in BATLAB and exported in ASCII format for further analysis. Spike counts represent the number of spikes per 32 repetitions of the stimulus. For a small number of neurons, 64 or 75 stimulus repetitions were presented. The selectivity index across calls was calculated as (n_0_ /(n_*t*_ –1))^∗^100, where n_0_ is the number of calls not evoking a response, and n_*t*_ is the total number of presented calls (Hurley and Pollak, 2005; similar index in Mayko et al., 2012). Responses across calls based on spike rate were compared using the formula: ($\sum_{i=1}^{n}$(1–min/max))/n)^∗^100, where *n* = number of pairwise comparisons of the minimum versus maximum values for the two among calls (excluding comparisons with zero as denominator). With this measure, a score of 100% would still represent the response to a single call, while a score of 0% would represent an identical spike number in the response to all five calls. We also compared responses across calls based on spike number in a preference index, using the formula: (($\sum_{i=1}^{n}$(1–min/max))/n)^∗^100, where *n* = number of pairwise ratios of the minimum versus maximum values for the two among calls, (excluding pairs in which both values were zero). With this measure, a score of 100% would still represent the response to a single call, while a score of 0% would represent an identical spike number in the responses to all five calls.

Statistical comparisons were made using Statistica software (TIBCO, Palo Alto, CA, United States). Comparisons of spike count and peak numbers across calls were performed with repeated measures ANOVAs to control for different baseline firing rates in different neurons. Effects of 8-OH-DPAT were assessed using baseline and drug values for the same calls in the same neurons before and during drug application, with call type as a categorical factor. Pearson’s correlations were used to assess the similarities of responses to different pairs of calls across neurons. A repeated measures ANOVA was used to compare the effects of 8-OH-DPAT against a background of no drug application versus NAD-299 application. To do this, the change in spikes in 8-OH-DPAT relative to the preceding baseline condition were compared to the change in spikes during the combination of 8-OH-DPAT and NAD-299 relative to the preceding application of NAD-299 alone. This comparison was made only for neurons in which recordings were made in the baseline, 8-OH-DPAT, NAD-299, and NAD-299 + 8-OH-DPAT conditions. A factorial ANOVA was used to assess whether response features that remained or disappeared in 8-OH-DPAT differed in peak spike density. Only neurons that showed more than one response peak were used in this analysis, and peak spike density values normalized to the largest peak for a given neuron were used. Otherwise, every response feature was treated as an independent value. Tukey’s honestly significant different (HSD) was used as a *post hoc* test of differences within significant statistical models. The standard error of the mean (s.e.m.) was used to report variation in statistical groups in the text and figures.

Spike density functions were generated following the algorithm of Shimazaki and Shinomoto (2010). This approach allows kernel size to be optimized over time in order to achieve the best fit to local spikes. Spike density functions were generated by a custom script in Matlab by author SN (MathWorks, Natick, MA, United States). Spike density functions were constructed for set of 30 neurons which showed robust responses to calls, low levels of spontaneous activity, and which were recorded in both baseline conditions and in the presence of 8-OH-DPAT.

## Results

### IC Neurons Respond Broadly to Female BBVs

We presented a set of five recorded female broadband vocalizations (BBVs) that varied in duration, amplitude envelope, and spectrotemporal structure to 15 anesthetized adult male CBA/J mice (Figure 1). High-resistance glass micropipette electrodes were used to extracellularly record sound-evoked responses from 77 single neurons in the inferior colliculus. Responses to tones at a range of frequencies and intensities were also recorded in the same neurons. BBVs have a power spectrum biased toward low frequencies, although some harmonics extend into higher frequencies (Figure 1). Data were collected from neurons that showed a response to at least one of the BBVs. Because of this, the population of neurons responding to calls was biased toward lower frequencies. Neurons with response to tones (*n* = 64), had CFs in the range 6–55 Hz (mean: 21.3 ± 2.5 KHz).

**FIGURE 2:**
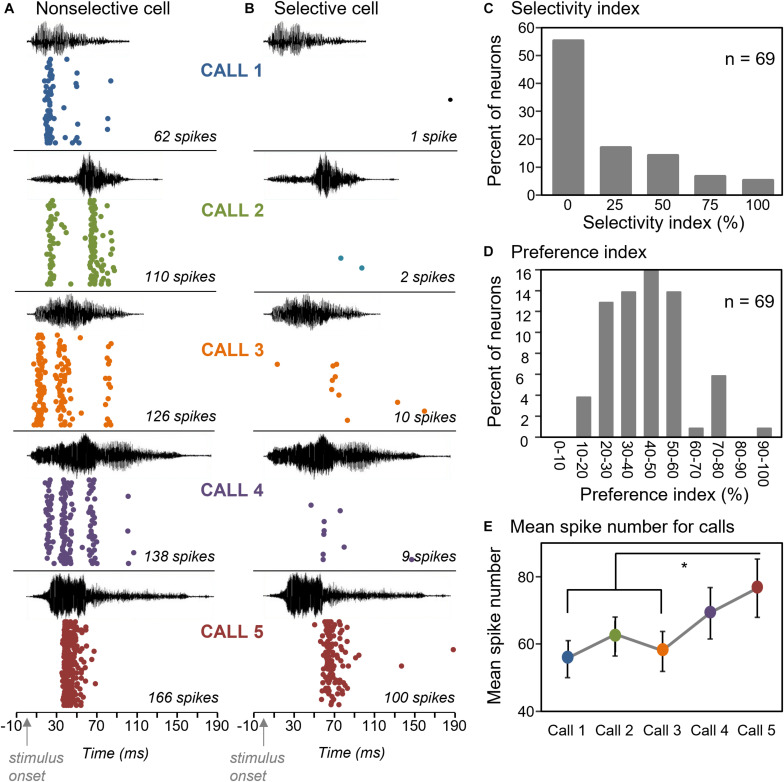
Selectivity of IC neurons for the five BBVs. **(A)** Raster plots of a neuron that responded to all five BBVs. **(B)** Raster plot of a neuron that responded robustly only to Call 5. **(C)** Selectivity index across a group of 69 neurons illustrating a low degree of selectivity, with most neurons responding to all calls. **(D)** Preference index, showing the mean per cent difference in spiking among pairs of calls across a group of 69 neurons. **(E)** Mean spike counts for responses across calls show significant differences, with Calls 1 and 3 evoking fewer spikes than Call 5. *p < 0.05.

We used a selectivity index to quantify how broadly neurons responded above a threshold value to the five calls. The selectivity index is (n_0_ /(*n*_*t*_ –1))^∗^100, where n_0_ is the number of calls not evoking a response, and n_*t*_ is the total number of presented calls (Hurley and Pollak, 2005; similar index in Mayko et al., 2012). With this index, the response of a neuron to a single call out of five would create a selectivity of 100%, while a response to all five calls would create a selectivity of 0%. The threshold value that we used to identify a response was a count of 20 spikes over 32 stimulus repetitions, or a response rate of 0.625 spikes/stimulus. This index was calculated for 69 neurons that showed a response to at least one call. Figure 2 shows neurons with different selectivity index values; one that responded strongly to all five calls and had a selectivity index of zero (Figure 2A) and one that responded only to one of the five calls and had a selectivity index of 100% (Figure 2B). Across the population of neurons, 55.1% of neurons showed a selectivity index of zero, 31.9% of neurons had a selectivity index of 25–50%, and the remaining 13% of neurons had a selectivity of 75–100% (Figure 2C). Thus, most neurons responded above a threshold value to most of the five BBVs.

Even for neurons that responded to multiple calls, there was variation in the numbers of spikes in responses to different calls (Figure 2A). We also compared responses across calls based on the total spike number in response to BBVs using a preference index with the formula: ($\sum_{i=1}^{n}$(1–min/max))/n)^∗^100, where *n* = number of pairwise comparisons of the minimum versus maximum values for the two among calls. With this measure, a score of 100% would still represent the response to a single call, while a score of 0% would represent an identical spike number in the responses to all five calls. The majority of neurons showed a preference index of 60% or less (Figure 2D), with an average preference index of 42.3 ± 2.0%. This value indicates that there was considerable variation in the intensity of the responses to different calls, even when a neuron responded to all of them.

To assess the sources of heterogeneity in spike numbers, we examined variation in spike number both across neurons, and across calls. Unsurprisingly, different neurons showed significantly different spike numbers, when responses to all five calls were considered [one-way ANOVA on spike counts with cell identity as a categorical factor, *F*_(68,276)_ = 11.36, *p* < 0.001]. Emphasizing the differences among neurons, the response to one call significantly correlated with the responses to the remaining four calls across the neural population (Pearson’s correlations, *p* < 0.00001 for all pairwise correlations among calls; Table 1). That is, the response of a neuron to a given call predicted it’s response to other calls. However, when we accounted for the strong effect of neuron identity by using a repeated measures approach, we found that different calls also evoked significantly different neural responses [repeated measures ANOVA with call type as a within-subjects factor; *F*_(4,272)_ = 5.19, *p* < 0.001]. Call 5 evoked the largest mean response, which significantly differed from responses evoked by both call 1 and call 3 (Figure 2E, Tukey’s HSD *post hoc* test; *p* < 0.001 for call 1 versus call 5 and *p* = 0.004 for call 3 versus call 5). Although the cause of the higher spike count is not clear, features of Call 5 such as the relatively high-intensity and sharp risetime of the first portion of the call, or its prolonged duration, could have contributed to high spike counts. These results show that spike number was influenced by both the identities of neurons and by call type.

**TABLE 1:** Pairwise *r*^2^ values for Pearson’s correlations between spike numbers for different call types across neurons.

	Call 2	Call 3	Call 4	Call 5
Call 1	0.46	0.62	0.52	0.44
Call 2		0.53	0.47	0.30
Call 3			0.75	0.55
Call 4				0.57

### Response Features Characterize Spiking Patterns Over Time

**FIGURE 3:**
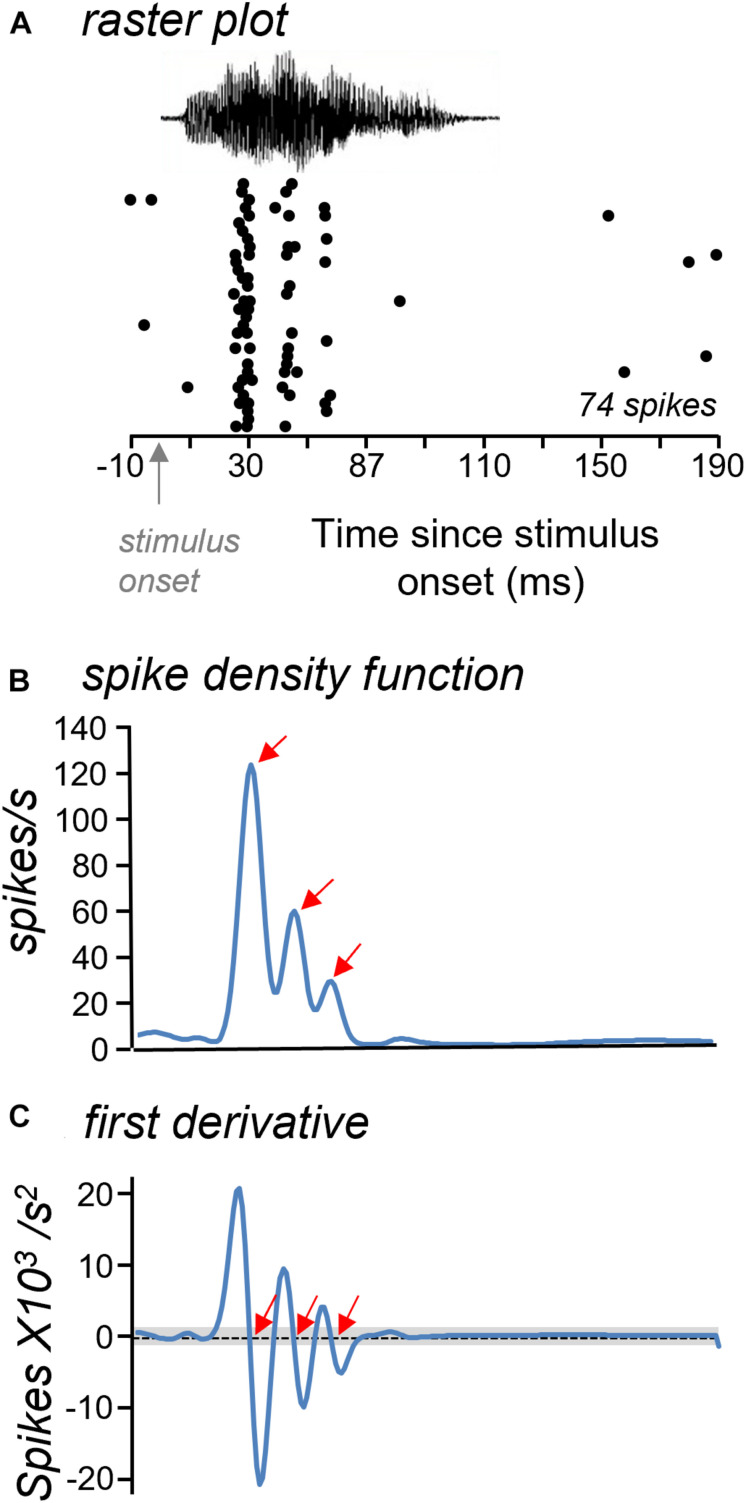
Approach for measuring response features. **(A)** Raster plots were used to generate spike density functions. **(B)** Spike density function with three clear peaks (arrows) illustrating periods of high spike probability. **(C)** Response features were identified from the first derivative of the spike density functions as null crossings that were higher and lower than the threshold criterion (gray bar). Red arrows mark identified response features. The time axis is the same in all plots.

Although neurons showed little difference in whether they responded to most calls, they differed in the patterns of response over time. The spike trains of most neurons were not evenly distributed over the timecourse of the BBV playback. Spike trains were often characterized by distinct time bins that had high spike probabilities (see Figure 2A). We called these localized peaks “response features.” Response features were quantified in a subset of 30 neurons with robust spike trains by generating smoothed spike density functions from raster plots of responses to BBVs (Figures 3A,B; Shimazaki and Shinomoto, 2010). Response features were identified based on the first derivative of the spike density function (slope) as null crossings with preceding accelerations and following decelerations of at least 1 unit in the first derivative function (Figure 3C). This ensured a uniform classification of response features. Numbers of response peaks ranged from as few as one to as many as seven for responses to single calls, with a mean of 2.37 ± 0.10 response features across all cells and all calls. Different calls also evoked significantly different numbers of response features [repeated measures ANOVA, *F*_(4,88)_ = 4.55, *p* = 0.002], with Call 4 evoking significantly more response features than either Call 1 or Call 3 (Tukey’s HSD, *p* = 0.002 for Call 1 versus Call 4, and *p* = 0.046 for Call 3 versus Call 4).

**FIGURE 4:**
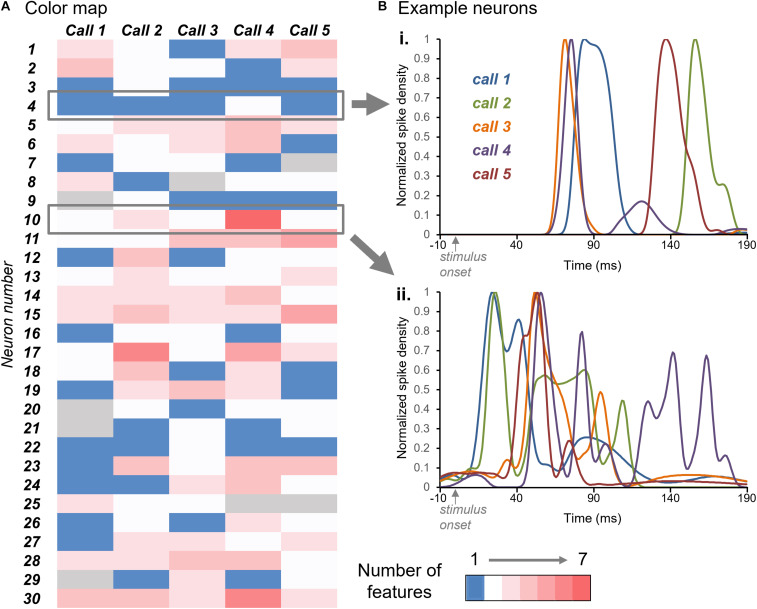
**(A)** Color map of numbers of response features for the responses of 30 neurons (*y*-axis) across the five BBVs (*x*-axis). Blue indicates single response features and white through red indicate increasing numbers of features, up to seven. Gray indicates the lack of response to a specific call. **(B)** Normalized spike density functions for two neurons indicated by the gray boxes in **(A)**. Spike density functions for the responses to all five calls are plotted together. For the neuron in **(i)**, the number of response features was one for four of the calls, and two for one of the calls. For the neuron in **(ii)**, the numbers of response peaks across calls ranged from two to seven.

The color map of Figure 4A illustrates the numbers of response features in the responses to each call (columns) by the group of 30 neurons (rows). Blue indicates a single response feature for the response of a given neuron to a given call, while white through dark pink indicate increasing numbers of response features (see color key). The color map illustrates a range of patterns across neurons. Some neurons showed similar numbers of response features across all five calls; for example, neuron #4 showed single response features to Calls 1, 2, 3, and 5, and two response features to Call 4 (Figure 4Bi). In contrast, neuron #10 showed a range of response features across calls, from two for Calls 1,3, and 5 to seven for Call 4 (Figure 4Bii).

**FIGURE 5:**
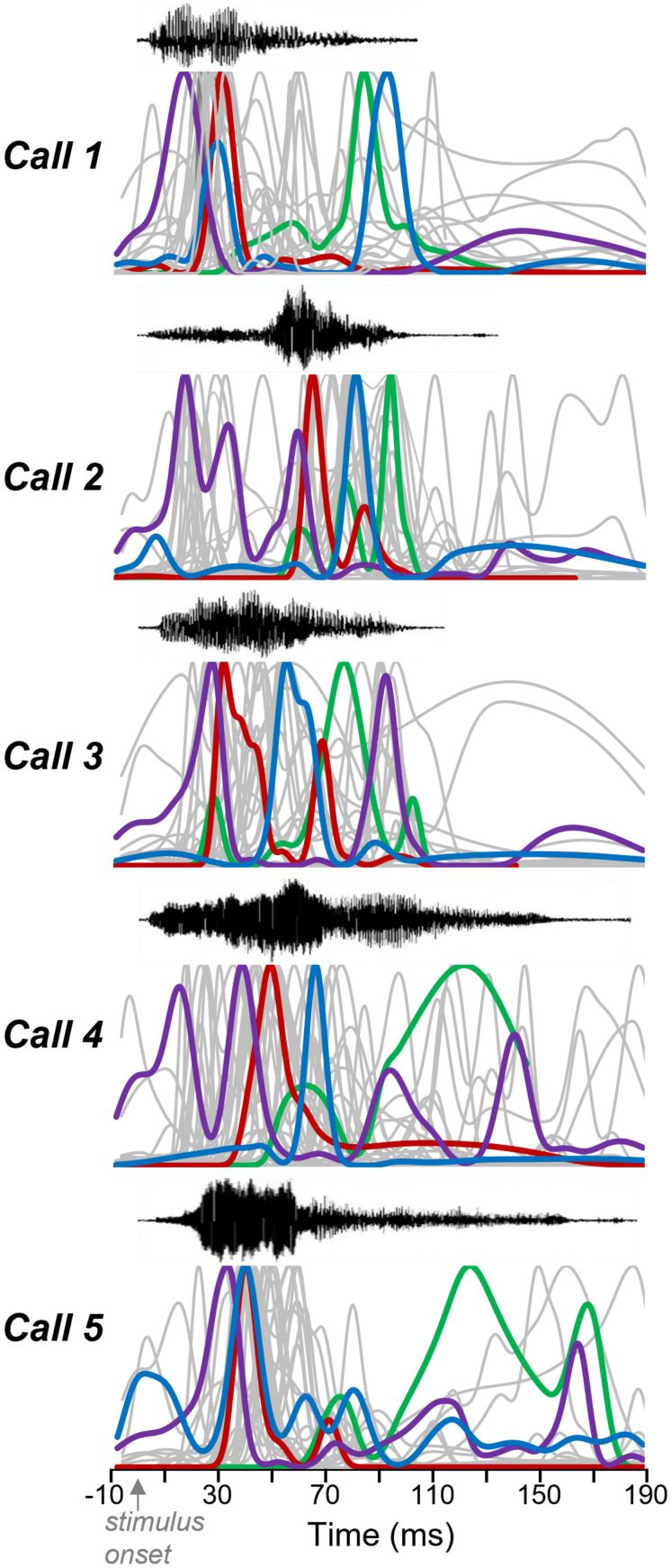
Response features vary among neurons and calls. Oscillograms (top) and normalized spike density functions for 30 neurons across five calls. Specific colors represent spike density functions of single neurons with a range of peak response times. A given color represents responses of the same neuron across calls. Gray spike density functions are from the remaining 26 neurons.

The number and timing of response features varied across neurons and also within neurons, across calls. These patterns are both illustrated in Figure 5, showing normalized spike density functions for each individual neuron, to facilitate a visual comparison among neurons. Four selected neurons are depicted as colored traces, with the same color representing the responses of the same neurons across calls. Gray traces represent the spike density functions of the remainder of the sample of 30 neurons. Across calls, the largest response features for single neurons occurred at different times. This is illustrated by the varying timing of the spike density functions of the color-labeled neurons across Calls 1–5. Single neurons may show some consistency in the location of their peaks across calls. For example: the neuron indicated in purple tended to respond relatively soon after the onset of calls, while the neuron indicated in green tended to respond relatively late in the spike train. Even so, all four example neurons showed responses that varied in timing and in the numbers of response features across the five calls. Some neurons even responded after calls had ended, as for some of the gray response peaks seen for Call 2. This pattern is consistent with the rebound from inhibition exhibited by some IC neurons.

### Pharmacological Manipulation of 5-HT1A Receptors

We assessed the effect of pharmacological manipulation of the 5-HT1A receptor, which is expressed in the IC (Thompson et al., 1994; Peruzzi and Dut, 2004; Smith et al., 2014), on responses to BBVs in 59 neurons. Neural responses were recorded before, during, and when possible after the iontophoresis of the 5-HT1A agonist 8-OH-DPAT. The suppressive effects of 8-OH-DPAT on stimulus-evoked responses in the IC have been reported previously in several studies (Hurley, 2006, 2007; Castellan Baldan Ramsey et al., 2010). Although 8-OH-DPAT is a commonly used agonist for the 5-HT1A receptor, it also activates the 5-HT7 receptor, which is functionally expressed in the auditory system (e.g., Tang and Trussell, 2015, 2017). To assess the selectivity of 8-OH-DPAT, we therefore combined the application of 8-OH-DPAT with the iontophoresis of NAD-299, a 5-HT1A antagonist. Because the effects of NAD-299 on IC neurons have not to our knowledge been reported, we iontophoresed this drug both alone and in combination with 8-OH DPAT. Our objectives were to (1) measure the effects of NAD-299 at increasing iontophoretic currents, as has previously been accomplished for 8-OH-DPAT, and (2) assess whether NAD-299 counteracted the suppressive effects of 8-OH-DPAT. Due to the difficulty of holding single cells for extended periods of time, not all of these objectives were achieved in the same neurons. In total, NAD-299 was applied to 40 neurons (alone: *n* = 35; +8-OH-DPAT: *n* = 25), and 8-OH-DPAT was applied to 46 neurons (alone: *n* = 46; +NAD-299: *n* = 25).

In contrast to 8-OH-DPAT, the effects of NAD-299 iontophoresis did not correspond linearly to increasing iontophoretic current. NAD-299 sometimes had a bimodal effect on firing rates, such that spike counts increased relative to control at low iontophoretic currents (the opposite of the effect of 8-OH-DPAT), but decreased at higher currents. An inverted U-shaped dosage function has previously been reported for this drug, with lower doses acting more selectively on 5-HT1A receptors, and higher doses activating α1 and β adrenoceptors (Johansson et al., 1997; Martin et al., 1999; Ross et al., 1999). To account for the possibility that NAD-299 affected multiple targets, we compared the effects of 8-OH-DPAT relative to a baseline of no drug application and relative to a baseline of NAD-299. In this way, even if NAD-299 affected additional targets, as long as it also blocked the 5-HT1A receptor, we reasoned that it should reduce the suppressive effects of 8-OH-DPAT. For this approach to work, it was important to compare the effects of 8-OH-DPAT on a baseline of no drug application versus a baseline NAD-299 iontophoresis in the same neurons. We accomplished the entirety of these sequential conditions in a group of 20 neurons. Because this pharmacological goal did not require responses to BBVs, we quantified this comparison for responses to the stimulus producing the largest effect for a given neuron, whether this was a BBV, a tone at the BF, or NBN.

**FIGURE 6:**
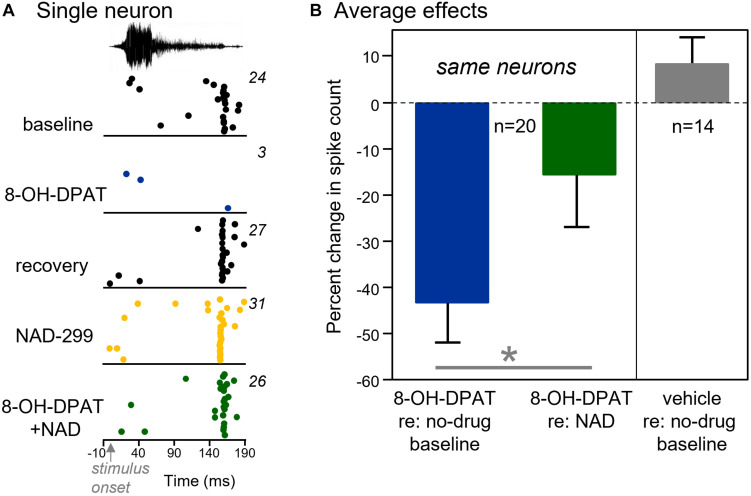
Activation of the 5-HT1A receptor reduces the numbers of sound-evoked spikes. **(A)** For a single neuron, the 5-HT1A agonist 8-OH-DPAT abolishes the response to a BBV. Iontophoresis of the 5-HT1A antagonist NAD-299 precluded the reduction in the number of spikes when it was iontophoresed before 8-OH-DPAT. The BBV was call 5 (see Figure 1), played at 10 dB above threshold. **(B)** *Left panel:* 8-OH-DPAT reduced the numbers of spikes significantly less on average when applied in the presence of NAD-299. *Right panel*: Application of vehicle solutions slightly increases but does not significantly alter the numbers of sound-evoked spikes relative to a no-drug baseline. *p < 0.05.

This outcome of this strategy is illustrated in Figure 6A, which shows raster plots of a single neuron to Call 5 in the baseline condition and during iontophoresis of 8-OH-DPAT, during a recovery, and during iontophoresis of NAD-299 and a combination of NAD-299 and 8-OH-DPAT (five conditions in total). For this neuron, 8-OH-DPAT showed a typical suppressive effect and recovery. NAD-299 did not alter the neuron’s response alone, but precluded the suppressive effect of 8-OH-DPAT when the two drugs were iontophoresed simultaneously. Across the group of 20 neurons that were treated with these drug combinations, 8-OH-DPAT decreased spike counts significantly less in the presence of NAD-299, [Figure 6B left panel; repeated measures ANOVA, *F*_(__1,19__)_ = 5.08, *p* = 0.036]. That is, the suppressive effect of 8-OH-DPAT was greater when compared to a baseline of no iontophoresis versus a baseline of NAD-299 iontophoresis. This finding is consistent with NAD-299 precluding the effect of 8-OH-DPAT. In contrast to the effects of iontophoresed drugs, controls consisting of the iontophoresis of control solutions (1 M NaCl: *n* = 8; 10 mM NaBr in 1 M NaCl: *n* = 6) did not significantly alter spike counts [Figure 6B right panel; repeated measures ANOVA, *F*_(__1,13__)_ = 1.19, *p* = 0.294].

### 8-OH-DPAT Suppresses Spiking and Reduces Response Features

As previously reported for other types of auditory stimuli, the iontophoresis of 8-OH-DPAT decreased the number of spikes in response to BBV playback. These effects were highly significant [repeated measures ANOVA with call type as a within-subjects variable; for drug effect *F*_(1,89)_ = 116.57, *p* < 0.001; for call type *F*_(4,189)_ = 0.65, *p* = 0.63; for drug × call interaction, *F*_(4,189)_ = 0.26, *p* = 0.90]. Suppressive effects were additionally not different across call types (Tukey’s HSD, *p* < 0.01 for baseline versus 8-OH-DPAT comparisons for each call type).

**FIGURE 7:**
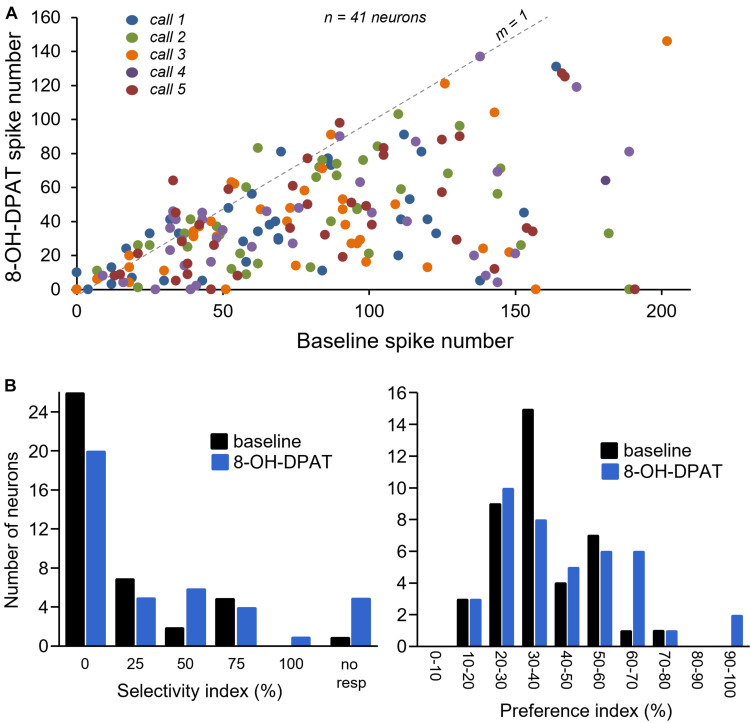
**(A)** Comparison of numbers of spikes in response to specific calls in the baseline (no drug application) versus during 8-OH-DPAT application. Symbols below the line with a slope of 1 represent a suppression of spikes during drug application. Different colors represent responses to different calls. **(B)** Selectivity index and preference index in a subset of 41 neurons in baseline and during exposure to 8-OH-DPAT.

Figure 7A illustrates spike counts in the no-drug baseline versus during iontophoresis of 8-OH-DPAT, for all calls in 41 neurons with robust call responses for which 8-OH-DPAT was successfully applied. The line with a slope of one marks where points would fall if spike numbers were identical in each condition; points below this line indicate decreased spike numbers during 8-OH-DPAT application. Although spike counts increased slightly for some neurons, the effects of 8-OH-DPAT were almost exclusively suppressive. Along with the decrease in spike numbers, 8-OH-DPAT shifted the population distribution for both the selectivity index and the preference index, in the subset of 41 neurons exposed to 8-OH-DPAT (Figure 7B). The population became more selective for calls (selectivity index of 13.6 ± 4.3% in baseline versus 24.3 ± 5.2% in 8-OH-DPAT), and showed a higher preference index (preference index of 37.7 ± 2.2% in baseline versus 43.0 ± 3.1% in 8-OH-DPAT). However, only the increase in call selectivity was significant [repeated measures ANOVA for baseline versus 8-OH-DPAT selectivity index, with a category for responses to no calls, *F*_(1,40)_ = 7.90, *p* = 0.008; repeated measures ANOVA for baseline versus 8-OH-DPAT preference index *F*_(1,40)_ = 3.47, *p* = 0.07].

We next assessed the effect of 8-OH-DPAT on the patterns of response features. In parallel with its suppressive effects on spike count, 8-OH-DPAT reduced the numbers of response features in the 30 neurons in which response features were assessed [repeated measures ANOVA with call type as a categorical factor, for drug effect *F*_(1,137)_ = 57.39, *p* < 0.001; for call type *F*_(4,137)_ = 1.92, *p* = 0.11; for drug × call interaction, *F*_(4,187)_ = 0.40, *p* = 0.81]. In contrast to the effects of 8-OH-DPAT on spike counts, the significant reduction in response features did not occur for all call types (Tukey’s HSD, *p* = 0.32 for Call 1, *p* = 0.003 for Call 2, *p* = 0.08 for Call 3, *p* = 0.01 for Call 4, *p* = 0.002 for Call 5). The lack of significant reduction in response features for Calls 1 and 3 corresponds to the lower numbers of baseline features for these calls (see above).

**FIGURE 8:**
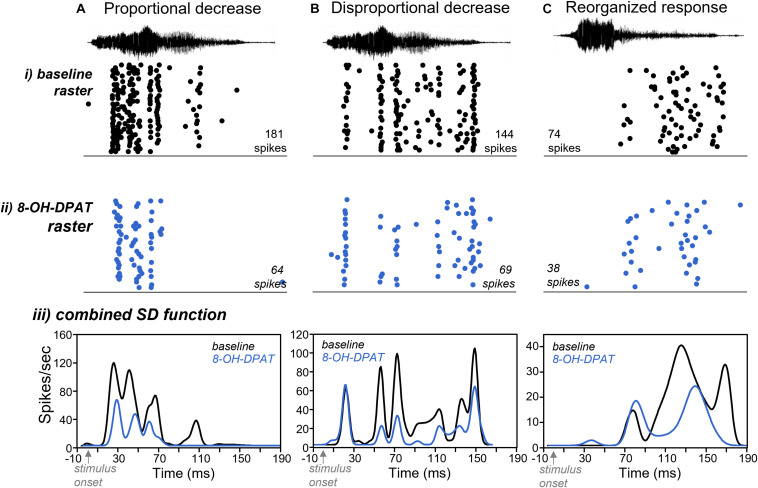
Effects of 8-OH-DPAT on response features. **(A)** Raster plots of the responses of a neuron in **(i)** the baseline condition and **(ii)** during iontophoresis of 8-OH-DPAT. **(iii)** Spike density functions derived from the rater plots in **(i)** and **(ii)**. The neuron in **(A)** showed proportionally similar suppressions of all response features, with the loss of the smallest feature. Call 4 evoked this response. **(B)** Neuron showing selective decreases of response features in the middle of its spike train. Call 4 evoked this response. **(C)** Neuron showing a reorganization of its spike train in the presence of 8-OH-DPAT. Call 5 evoked this response. Time scale is the same for all panels.

We further explored which types of response features were most likely to disappear. Figure 8 shows three general patterns of the effect of 8-OH-DPAT on response features: proportionate, disproportionate, and reorganizing. The most common of these was a proportionally consistent decrease in the sizes of response features, with the loss of some features. This response pattern was defined as having response features during the application of 8-OH-DPAT that had the same rank order of peak size as in the baseline. Neurons for which all response features except for the largest disappeared, and for which all response peaks including the largest disappeared, were counted in this category. For example, 8-OH-DPAT caused a proportional decrease in the sizes of the three largest of the response features of the neuron in Figure 8A, and the disappearance of the smallest response feature. A second type of pattern was the selective loss of some response features but the maintenance of others, to create a disproportionate effect. This type of effect was defined as a reversal in the rank order of peak size in the presence of 8-OH-DPAT relative to baseline. However, even if relative peak sizes reversed, if the proportional effect of 8-OH-DPAT (peak sizes in 8-OH-DPAT relative to baseline) were within 20% of each other, responses were not included in this category. For the neuron in Figure 8B, 8-OH-DPAT caused a strong suppression of response features in the middle of the spike train, but relatively lesser effects on the response features at the end and particularly the start. Finally, a pattern seen in only a few neurons was the reorganization of the spike train so that response features occurred at different times (Figure 8C). This effect was defined as the appearance in 8-OH-DPAT of new peaks with peak latencies more than 10 ms from peaks in the no-drug baseline, as long as the new peaks did not occur within rising or falling time ranges of peaks in the baseline. Out of a total of 103 responses that had more than one peak in the baseline in response to all five calls, 74 (71.8%) showed proportionate effects of 8-OH-DPAT, 19 (18.4%) showed disproportionate effects, and 10 (9.7%) showed reorganizations. Out of the 30 neurons, 14 showed only proportionate effects, although responses to many individual calls in this group had only one response peak, or showed no response peaks during 8-OH-DPAT iontophoresis. Of the 16 neurons that showed non-proportionate effect of 8-OH-DPAT on response peaks (disproportionate or reorganization), eight neurons only had one call with non-proportionate effects, three neurons had two calls with disproportionate effects, and five neurons had three calls with disproportionate effects. Thus, although most neurons showed mixed proportionate and disproportionate effects of 8-OH-DPAT across calls, a minority of neurons had a relatively high number of call responses that were disproportionate.

**FIGURE 9:**
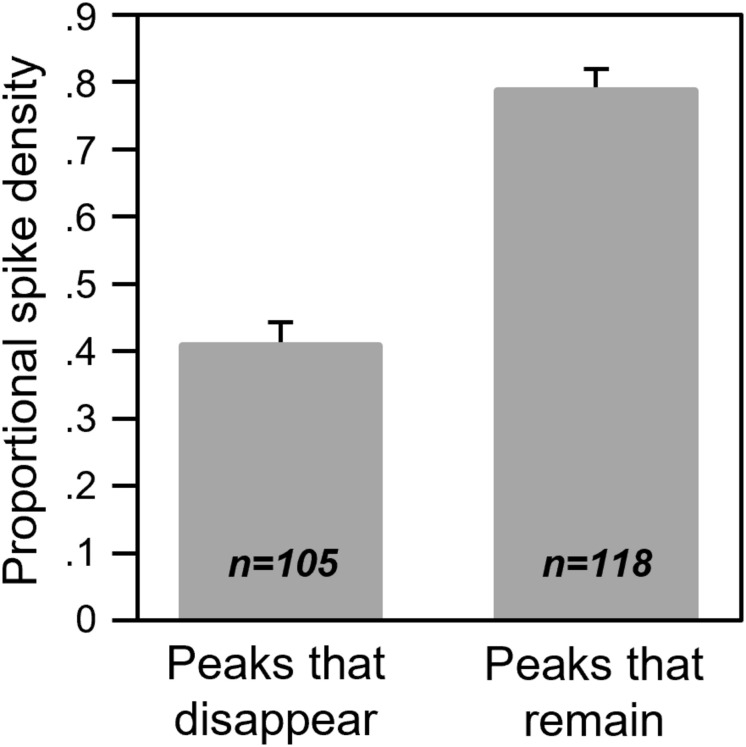
Proportional spike densities for response features that disappeared or remained during iontophoresis of 8-OH-DPAT. The peak spike densities of response features were normalized to the largest response feature in their respective spike trains.

Of these patterns, the most common proportionate effect (Figure 8A) suggests that the smallest response features are the most likely to disappear. To test this hypothesis, we compared the spike densities of response features that were lost during 8-OH-DPAT versus those that were maintained. To account for the variation in spike rates, we normalized the spike densities for each response feature to the spike density for the largest response feature in the spike train. Across the population of neurons, response features that disappeared were significantly smaller than their neighbors [factorial ANOVA for normalized peak height, with status (whether peaks were lost in 8-OH-DPAT or not) and call type as categorical factors: for status *F*_(1,209)_ = 97.3407, *p* < 0.001; for call type *F*_(4,209)_ = 0.6214, *p* = 0.65; for status × call type *F*_(4,209)_ = 1.84, *p* = 0.12]. Average proportional sizes for all peaks that were lost in 8-OH-DPAT versus not lost are shown in Figure 9.

## Discussion

Serotonin is a neuromodulator that conveys information on social context to auditory regions and modulates auditory processing (Wang et al., 2008b; Hall et al., 2011; Hurley and Sullivan, 2012; Hanson and Hurley, 2014; Keesom and Hurley, 2016). Although serotonin influences the responses to ultrasonic acoustic signals in the auditory system (Hurley and Pollak, 2005), whether these effects extend to audible vocal signals, and the roles of specific receptor pathways in these effects, have not been explored. In the current study, we measured how neurons in the IC respond to five exemplars of a type of audible mouse vocalization, BBVs, and how the 5-HT1A receptor pathway alters responses to this type of call. We found that IC neurons respond to BBVs with characteristic local periods of high spike probability that we called “response features.” Local activation of the 5-HT1A receptor within the IC had suppressive effects on BBV-evoked spike numbers that were similar to previously reported effects on responses to tones (Hurley, 2006, 2007; Castellan Baldan Ramsey et al., 2010). However, 5-HT1A-gated suppression interacted with response features to change the temporal patterns of neural responses to BBVs. In the following discussion, we compare the BBV responses we measured to previous reports of responses to acoustic signals in the IC, speculate on the mechanisms of the effects of 5-HT1A receptors, and frame the influence of 5-HT1A receptors in the context of additional serotonin receptor types and of the behavioral conditions that cause elevated serotonin in the IC.

### How IC Neurons Respond to BBVs

Most work on the behavioral and neural responses to acoustic signals in mice has focused on USVs, an ultrasonic signal type produced by males and females in both same-sex and opposite-sex interactions (e.g., Nyby, 1983; Holy and Guo, 2005; Holmstrom et al., 2010; Hanson and Hurley, 2012; Grimsley et al., 2013; Neunuebel et al., 2015; Warren et al., 2018). Similar to USVs, BBVs, which contain human-audible harmonics (Lupanova and Egorova, 2015; Finton et al., 2017), are produced across a range of behavioral contexts. BBVs are made by both sexes during non-social distress and same-sex aggression (Irwin et al., 1971; Matthews et al., 2008; Lupanova and Egorova, 2015; Finton et al., 2017). During opposite-sex interactions, BBVs are produced largely by females as they direct kicks and lunges at males, but are also produced as females allow males to mount (Wang et al., 2008a; Sugimoto et al., 2011; Finton et al., 2017). From the perspective of a male listener, BBVs might therefore have different behavioral salience at different phases of an opposite-sex interaction. Male perception of BBVs also depends on context. Males approach speakers broadcasting BBVs less when they are paired with the odor of a predator than with female urine (Grimsley et al., 2013).

Since USV responses have been more often recorded than BBV responses, it is useful to compare the two. Given the extreme differences in the structures of BBVs and USVs, one might predict a segregation in the responses to USVs versus BBVs to neurons with low and high CFs, respectively. However, this may not be the case. Although IC neurons that respond to calls with low frequency harmonics often have at least one harmonic that falls within the frequency tuning curve, this is not always true for USVs (Portfors et al., 2009). CFs of IC neurons responding to USVs are often lower than predicted, suggesting that there may be substantial overlap in the CFs of neurons responding to BBVs and USVs. A second comparison is in the selectivity of neural responses for USVs and BBVs. Responses to USVs in the IC have from high to moderate levels of selectivity across studies in bats and mice (Hurley and Pollak, 2005; Portfors et al., 2009). In the current study, BBV responses were strikingly non-selective, with over 72% of neurons responding to four or five BBVs. The fact that mice in the current study were anesthetized could have contributed to this extreme lack of selectivity, but the different structures of BBVs and USVs could also potentially contribute to differences in the selectivity of responses to these two signals. USVs typically consist of single or at most two widely separated harmonics (Holy and Guo, 2005; Hanson and Hurley, 2012). In contrast, BBVs are composed of many harmonics that can extend into the ultrasonic range (Figure 1). This broadband structure makes it more likely that BBVs will evoke responses from neurons across a broad range of CFs, and that single neurons will respond to different BBVs. This possibility is supported by the auditory responses of IC neurons in another rodent species, guinea pigs, in which responses to human-audible vocalizations have been recorded. Guinea pigs produce multiple low-frequency harmonic calls that differ significantly in temporal structure (Šuta et al., 2003). Remarkably similar to the current study, 55% of neurons respond to every stimulus type presented in the guinea pig IC (in comparison to 55.1% of neurons in the current study).

Although IC neurons were not selective for BBVs at the level of responses to whole calls, temporal patterns in response to the same calls appeared to be relatively more selective, in that they varied across neurons and across calls (Figures 2, 5). We characterized these patterns by defining “response features,” as temporal regions of high spike probability surrounded by areas of lower probability. In the current study, response features were likely to be driven by both the specific spectrotemporal characteristics of calls and the intrinsic properties of neurons. For example, some of the neurons in Figure 5 responded near the start of calls with pronounced onsets, such as Calls 1 and 5. Likewise, some neurons responded in regions of calls characterized by deterministic chaos, which is structured noise driven by non-linear vocal fold vibration (Figure 8C; Fitch et al., 2002; Lupanova and Egorova, 2015). The burst of spikes observed shortly after the end of BBVs in some neurons is consistent with rebound firing, an intrinsic property of some IC neurons (Figure 5). With a playback sample of five calls, it is not possible to definitively state which characteristics of calls triggered specific response features. However, the general observation that IC neurons are selective for specific spectrotemporal aspects of stimuli has been observed in multiple studies using systematically varying stimuli to generate spectrotemporal receptive fields (Andoni et al., 2007; Rodríguez et al., 2010; Chen et al., 2012; Park et al., 2021). In relation to the current study, the response features that vary across neurons and across calls suggest that the neural coding of BBVs is more selective when considering response features than responses to whole calls.

### 5-HT1A Receptor

For most IC neurons, activation of the 5-HT1A receptor by 8-OH-DPAT exerted a suppressive gain control. This effect is similar to previous work in the IC using synthesized stimuli such as tones and FM sweeps (Hurley, 2006, 2007). Spike suppression by 8-OH-DPAT is also consistent with the effects of 5-HT1A receptors in other brain regions, where these receptors also mediate response suppression (Albert and Vahid-Ansari, 2019). The 5-HT1A receptor is generally expressed somatodendritically or in axon hillocks, and acts via G-protein coupled inward rectifier potassium (GIRK) channels to suppress firing in a number of neuron types (Albert and Vahid-Ansari, 2019). In the medial superior olive (MSO), an auditory brainstem nucleus, 5-HT1A receptors are expressed in the axon initial segment, and regulate neural output by affecting spike probability (Ko et al., 2016). In the IC, the subcellular location of the 5-HT1A receptor has not been assessed, but the fact that most neurons showed roughly uniform effects of 8-OH-DPAT for different response features is consistent with expression by the IC neurons being recorded. The differential effects of 5-HT1A activation on specific response features in some neurons (Figure 8B) also suggest the possibility that 5-HT1A receptors could influence the activity of presynaptic IC neurons with response profiles different from those of the neurons being recorded.

Even for neurons with proportionally similar effects of 8-OH-DPAT on different response features, some response features were maintained while others disappeared. The smallest peaks were the most likely to disappear during 5-HT1A activation, consistent with an “iceberg”-like effect, in which suprathreshold responses are more selective than subthreshold responses (Rose and Blakemore, 1974). Neurochemical regulation of the strength of responses relative to threshold is an established mechanism for regulating the selectivity of IC neurons. For example, the manipulation of inhibition can alter primary response characteristics such as frequency tuning (Fuzessery and Hall, 1996; Xie et al., 2007; Wu and Jen, 2009), as well as more complex response characteristics including duration sensitivity (Alluri et al., 2016) or sensitivity to the velocity of frequency modulation (Gittelman and Li, 2011). For the neurons in the current study, the outcome of 5-HT1A-induced spike suppression was to alter the response profile at the level of single neurons, as they showed fewer response features. The disappearance of peaks could also potentially translate to a sparser and more selective population response, with fewer neurons responding to specific spectrotemporal call features.

### Other Serotonin Receptors

Because there are multiple types of serotonin receptor, the effects of 5-HT1A activation during serotonin release would depend on the context of other activated receptors. Members of five of the seven main families of serotonin receptor are expressed in the IC, and some of these families have more than one subtype (Hurley and Sullivan, 2012). Serotonin receptors have a wide range of effects on neural excitability and neurotransmission in the IC. In addition to spike suppression by the 5-HT1A receptor, the 5-HT2 receptor reduces the frequency and amplitude of GABAergic and glycinergic IPSCs (Wang et al., 2008b). Activation of the 5-HT1B receptor also has facilitatory effects that are consistent with the inhibition of GABAergic transmission, an effect precluded by prior activation of GABA_*A*_ receptors (Hurley et al., 2008).

There are several excellent examples of how other types of serotonin receptors interact with the 5-HT1A receptor in the auditory system. In the dorsal cochlear nucleus, activation of the 5-HT2A receptor increases the excitability of principal neurons (Tang and Trussell, 2015), while increasing the recruitment of feed-forward inhibitory inputs (Tang and Trussell, 2017). At the same time, 5-HT1A receptors decrease excitatory inputs to the principal neurons evoked by auditory nerve fiber stimulation (Tang and Trussell, 2017). Since inputs from multisensory pathways do not show these presynaptic changes, this suite of effects increases responses from multisensory pathways relative to auditory-specific pathways, gating these different sources of information. In pyramidal neurons from Layers II/III of the auditory cortex, 5-HT1A and 5-HT2A receptors reduce GABAergic transmission presynaptically and postsynaptically, respectively (García-Oscos et al., 2015). At the same time, 5-HT1A activation postsynaptically reduces the amplitudes of EPSCs (Cervantes-Ramírez et al., 2019).

In the IC, two receptor types in the 5-HT1 family, the 5-HT1A and 5-HT1B receptors, overlap in their effects on single IC neurons. Activation of these receptors have opposite and additive effects on the firing rates of IC neurons, but 5-HT1A activation has effects on spike timing that are dominant to those of the 5-HT1B receptor (Castellan Baldan Ramsey et al., 2010). These findings suggest that the two receptor types may interact in non-linear ways to influence patterns of neural responses. Although the interaction of serotonin receptor types at the microcircuit level has not been described in the IC, a working hypothesis is that the 5-HT1A receptor could suppress responses to inputs closest to threshold, and increase contrast with the selective disinhibition of presynaptic inputs by other types of serotonin receptor. Because most ascending auditory pathways make synaptic connections in the IC, these effects could filter information on BBVs en route to the auditory thalamus.

An important caveat to our findings is that they were generated in anesthetized subjects. Mice would typically be awake and interacting socially when perceiving calls from other mice, with a physiological state that is different from that of an anesthetized mouse. An alternative to the approach used in this study could have been working with awake restrained mice (e.g., Gourévitch et al., 2020). Although this would differ from an anesthetized state, this model would still not fully capture the physiology of a socially interacting brain. Recording multiunit responses in behaving mice would address this issue and allow for comparison between neural responses and behavior. However, the type of recording in the current study in which well-isolated single neurons are exposed to highly localized manipulation of specific receptor types, is difficult to achieve in moving subjects. Furthermore, recording in anesthetized mice does have some advantages, one of which is the relatively low level of endogenous serotonin release in the IC (Hall et al., 2010). In awake and behaving mice, serotonin levels fluctuate in different social contexts (Hall et al., 2011; Hanson and Hurley, 2014; Keesom and Hurley, 2016), so that the availability of 5-HT1A receptors could also fluctuate, potentially leading to different effects exogenous 5-HT1A activation across contexts. Anesthesia is also unlikely to change the basic suppressive effect of the 5-HT1A receptor, since suppressive effects of this receptor have also been reported in the IC in an awake bat model (Hurley, 2007). All in all, although the use of anesthetized mice may not fully represent the responses of auditory neurons to social signals, this approach can provide a view of the potential repertoire of specific receptor types on a local scale in influencing call responses, particularly if combined with additional studies in awake animals.

### Behavioral Context

In different vertebrate species, different neuromodulatory systems modify the responses to acoustic social signals in accordance with behavioral context. In auditory forebrain regions in female zebra finches, local introduction of norepinephrine or an α2 adrenergic agonist increases the signal-to-noise ratio for a range of stimuli including conspecific and heterospecific songs, in large part by decreasing spontaneous activity (Ikeda et al., 2015). These changes improve the accuracy of a stimulus classification algorithm, demonstrating improved encoding of stimuli during adrenergic activation.

A sensory region in which a feedback loop has been established between enhanced serotonin release triggered by a social signal and the effects of serotonin on signal processing is the electrosensory lobe (ELL) of brown ghost knifefish. This and other weakly electric fish species use self-generated electric organ discharges to navigate and communicate, via their electrosensory systems (Lissmann, 1951; Hopkins, 1988). Simulated conspecific electric signals cause rapid increases in serotonin in the ELL (Fotowat et al., 2016). Increased serotonin in turn selectively increases the excitability of the principal neurons in the ELL to simulated same-sex electric signals (Deemyad et al., 2013). Serotonin increases burst firing and sensitivity to fluctuations in the stimulus envelope, a change that is further reflected in enhanced behavioral responsiveness (Marquez and Chacron, 2020).

In mice, previous work describing serotonin release in the IC during opposite-sex interaction is helpful in framing the effects of serotonin on BBVs within this context. Serotonin levels in the IC of males interacting with female partners is inversely correlated with rejection, measured by the numbers of BBVs, made by female social partners (Keesom and Hurley, 2016). Although this finding alone would suggest that BBVs are not produced when serotonin levels are elevated (when females do not reject males), copious BBVs are also produced when females allow males to mount (Wang et al., 2008a; Finton et al., 2017). Furthermore, Calls 4 and 5, with longer durations and longer relative segments of DC, are similar to calls more likely to be produced during mounting behavior than during female rejection (Finton et al., 2017). Neural responses to BBVs, particularly those similar to Calls 4 and 5, in this behavioral subcontext might therefore be most subject to serotonergic modulation, with BBVs produced in the process of female rejection relatively unmodulated by serotonin. The serotonergic modulatory system could therefore contribute to the differential auditory processing of BBVs in different behavioral subcontexts. Whether these events in the IC can influence the perception of BBVs by males in these subcontexts is currently unknown.

## Data Availability Statement

The raw data supporting the conclusions of this article will be made available by the authors, without undue reservation.

## Ethics Statement

The animal study was reviewed and approved by the Bloomington Institutional Animal Care and Use Committee, Indiana University Bloomington, United States.

## Author Contributions

AG and LH performed the experiments and data analysis, and contributed to writing the manuscript. SN performed the data analysis and contributed to writing the manuscript. All authors contributed to the article and approved the submitted version.

## Conflict of Interest

The authors declare that the research was conducted in the absence of any commercial or financial relationships that could be construed as a potential conflict of interest.

## Publisher’s Note

All claims expressed in this article are solely those of the authors and do not necessarily represent those of their affiliated organizations, or those of the publisher, the editors and the reviewers. Any product that may be evaluated in this article, or claim that may be made by its manufacturer, is not guaranteed or endorsed by the publisher.
